# Support workers’ experiences of work stress in long-term care settings: a qualitative study

**DOI:** 10.1080/17482631.2019.1622356

**Published:** 2019-06-03

**Authors:** Karol J. Czuba, Nicola M. Kayes, Kathryn M. McPherson

**Affiliations:** a Centre for Person Centred Research, Auckland University of Technology, Auckland, New Zealand; b Health Research Council, Auckland University of Technology, Auckland, New Zealand

**Keywords:** Caregivers, qualitative research, occupational stress

## Abstract

**Background**: Support-workers’ performance and well-being are challenged by increasingly high workloads and poor working conditions, leading to high levels of occupational stress.

**Aims**: To explore the experiences of work stress for support-workers in New Zealand residential facilities.

**Design**: An Interpretive Descriptive study.

**Methods**: Data from ten (n = 10) support-workers were collected between December 2013 and June 2014, using semi-structured in-depth face-to-face interviews. Thematic analysis was used to identify key themes that captured participant reports of their experiences.

**Results**: Work stress was conceptualized by participants as being an everyday experience of having too much to deal with and feeling under constant pressure. It appeared to be a complex and fluid experience representing an inherent, dynamic tension between reasons to be a caregiver and the burden of caregiving. Participants highlighted a range of influencing factors (including lack of recognition, person and work context, and coping strategies), which may account for that fluidity.

**Conclusion**: The findings extend current knowledge about support-workers’ work stress by identifying the challenges relating to the lack of recognition of their role and expertize, the unintended consequences of person-centered care and the challenges faced by migrant support-workers.

## 1. Introduction

Healthcare systems around the world face a major challenge in providing care to a growing number of people living with long-term conditions (Blendon & DesRoches, 2003; Health Education England, 2015). Over 60% of hands-on care in long-term care settings is provided by support-workers (Health Education England, 2015), these being caregivers without formal qualifications, or with no recognition if those qualifications were gained outside the countries in which they are working. Their main duty is to assist frail older people, disabled and/or ill people with activities of daily living (Ravenswood, Douglas, & Teo, 2014). The demand for their services already exceeds the supply of available workers (King et al., 2013; Ravenswood et al., 2014) which contributes to an increased workload for this group.

It has been argued that increased workload leads to an increase in occupational stress (Dawson, Stasa, Roche, Homer, & Duffield, 2014; Halpin, Terry, & Curzio, 2017; Weiss & Lonnquist, 2012). This in turn has been shown to have a negative impact on staff turnover (Leiter & Maslach, 2009; Meeusen, Van Dam, Brown-Mahoney, Van Zundert, & Knape, 2011), job satisfaction (Farquharson et al., 2013; Zangaro & Soeken, 2007), health and well-being (Juthberg, Eriksson, Norberg, & Sundin, 2010; Orrung Wallin, Jakobsson, & Edberg, 2013), and on the quality of care (Zúñiga et al., 2015). Given global demand in the face of limited financial and human resources (Blendon & DesRoches, 2003), the workload is unlikely to decrease in the foreseeable future. Therefore, it is crucial to focus on developing evidence-based strategies that recognize the increasing demand on this population and help to alleviate the abovementioned issues.

Work stress has long been a focus in health and workplace management research. One of the most influential models of stress is the Transactional Model of Stress and Coping (Lazarus & Folkman, 1984). It defines stress as “a pattern of negative physiological states and psychological responses occurring in situations where individuals perceive threats to their well-being, which they may be unable to meet” (McIlveen & Gross, 1997). The model emphasizes the role of job situation, subjective perception and individual differences in how people experience stress. As such, it appears particularly relevant for studies exploring subjective perceptions and experiences, and has some congruence with research exploring work stress for support workers.

Research on support-workers’ work stress and its implications is scarce, with the support-workers usually being mixed in with other health workforce groups, e.g. nurses. Research that does exist has found that one of the major stressors for support-workers is the perceived lack of control (Clarke, 2001; Gustafsson, Norberg, & Strandberg, 2008; Hertting, Nilsson, Theorell, & Larsson, 2005; Mininel, Baptista, & Felli, 2011). This lack of control is reported to be experienced in decision-making situations, time management, and appears to contribute to feelings of powerlessness and helplessness. Support-workers reported feeling that no one at their workplace cared about their opinion (Mininel et al., 2011) and tended to believe that they were unable to change their situation (Gustafsson et al., 2008).Another important stressor reported to affect this workforce is work overload, i.e. when work demands exceed the caregivers’ ability to meet them (Clarke, 2001; D’Hondt, Kaasalainen, Prentice, & Schindel Martin, 2012; Hertting et al., 2005; Shaha & Rabenschlag, 2007; Zhang et al., 2011). Low “caregiver to resident ratios” and working with limited resources is a common finding, and is considered to be a current and future challenge for this workforce group (Clarke, 2001). Authors have found that lack of appreciation and respect is another significant work stressor (Gustafsson et al., 2008; Secrest, Iorio, & Martz, 2005; Zhang et al., 2011). This was observed across many different levels of support-workers’ work relationships, including with nurses, upper management, patients and their families. Support-workers report feeling that their work is not valued by other people in their organization with some even suggesting they have been treated as slaves (Secrest et al., 2005). Poor communication and teamwork are another factor contributing to these workers’ stress. The support-workers reported experiencing issues with upward communication, i.e. with their supervisors and managers, often leaving them with unresolved queries (Khalaf, Berggren, & Westergren, 2009; Zhang et al., 2011). Workers reported feeling lonely and abandoned due to lack of support from other colleagues (Khalaf et al., 2009), and feeling their supervisors did not care about their suggestions or feedback (Mininel et al., 2011). These findings highlight the role that a range of both individual-specific and context-specific stressors play in experiencing work stress for support-workers.

In recent years, studies have focused on predictors of occupational stress in support-workers and on associations with other important health outcomes (Hayes et al., 2012; Zúñiga et al., 2015). A literature review conducted by Hayes et al., 2012 focused on the causes and consequences of staff turnover in healthcare organizations. They found that excessive work-load played a significant role in staff turnover, particularly when combined with low job control and lack of team support. In a study of 4311 care workers (including registered nurses, licensed practical nurses, nurse aides), Zúñiga et al., 2015 found that better quality of care was associated with less stress experienced by the workers, and suggested more support in handling work stressors was required to promote quality care in nursing homes. It is clear, that the level of work stress experienced by support workers can have wide-ranging implications on the healthcare organizations and the quality of care they aim to deliver. However, to our knowledge, no studies have specifically explored the experience of work stress from the perspective of support-workers in a long-term care setting; with recent calls for research addressing this issue (Braedley, Owusu, Przednowek, & Armstrong, 2018).

Support-workers play one of the most important roles in the healthcare system (Health Education England, 2015; Pruitt, Canny, & Epping-Jordan, 2005) and their occupational stress impacts on them, their employers, and those for whom they care. There is a need to identify better ways of supporting these workers in coping with their work stress. However, before these can be developed, we require a more nuanced understanding of their experience of work stress to address it, and its impact on care quality, effectively. This study set out to explore the experience of work stress for support-workers, aiming to investigate two main questions:

- What are support-workers’ experiences of work stress?
- What do support-workers perceive as influencing their experience of work stress?

## 2. Methods

An Interpretive Descriptive Methodology was used (Thorne, 2016). This study was driven by the primary author’s interest in support workers in the context of inpatient healthcare facilities and the challenges they face. We wanted findings of this research to be easily understood by staff within those facilities and translated into practice. Interpretive Description has been designed to address such practice-driven investigations and as such was deemed appropriate to achieve these purposes (Hunt, 2009).

### 2.1 Sampling and recruitment

People were eligible to take part if they were support-workers without health professional qualifications recognized in the country (for example paid carers, nursing assistants, or similar), were currently working as a support worker in an inpatient healthcare facility (including but not limited to rest homes, residential rehabilitation facilities, private hospitals) and if they:

1. were 20 years or older;
2. were New Zealand (NZ) residents or citizens;
3. had at least one year of documented experience in their role; and
4. were working more than 20 hours per week.

This study used purposive and theoretical sampling, consistent with Interpretive Description methodology (Thorne, Kirkham, & MacDonald‐Emes, 1997). Purposive sampling was used initially aiming for diversity on key characteristics such as gender, ethnicity, experience in the role, immigration status. For example, as the majority of support-workers working at recruiting localities were women, there was a focus on recruiting a male participant. Attempts were also made to recruit people from a diversity of cultures and ethnicities, including those born outside NZ. Subsequently, theoretical sampling was used to allow exploration of key ideas evident in preliminary analysis and to test out early interpretations of data.

The first phase of recruitment for this study was through a flyer distribution at two local inpatient long-term rehabilitation facilities. However, only four workers volunteered to take part in the study. Hence, two unions representing NZ support-workers were contacted, and they agreed to post an advertizement in their monthly newsletters. This strategy proved successful with a further 17 people expressing their interest in the study.

Ongoing analysis suggested that the ninth and tenth interviews yielded no new categories or themes, indicating that further interviews were unlikely to have a significant impact on the findings of this study. Hence, recruitment stopped after ten participant interviews had been conducted.

### 2.2 Data collection

Data was collected between October 2013 and June 2014 using semi-structured in-depth face-to-face interviews, at participant’s preferred location (e.g. their home, workplace, café). The interviews were conducted by the primary author (KJC; male; overseas qualified physiotherapist who had worked as a support worker in NZ from 2010 to 2012, employed as a Research Assistant at the time of the first interview).

Interviews lasted between 30 to 60 minutes. Only the interviewer and participant were present at each interview. After a brief exchange preamble, participants were invited to recall and describe a stressful work event experienced in the last month. No definition of stress was provided to participants. Rather, they were asked to explain what stress meant to them at the outset of their interview. This was followed by further prompts exploring perceptions of support-workers work stress with respect to self and their work environment. After preliminary analysis of the first group of interviews, prompts were reviewed and refined in order to allow a deeper and more nuanced exploration of participant experiences. Each interview was audio-recorded and transcribed verbatim. Basic demographic information such as gender, age and ethnicity were also collected for the purpose of describing the sample. No repeat interviews were carried out and no member check occurred. All participants received a summary of findings. Field notes were made after each interview to capture the interviewer’s initial impressions.

### 2.3 Ethical considerations

This study received approval from the Auckland University of Technology Ethics Committee (reference number: 13/251).

The study was driven by the primary author’s (KJC) interest in formal carers working in the context of inpatient healthcare facilities and the challenges they face, largely driven by his experience of working in a support worker role as above.

Each participant received an information sheet explaining the study purpose. All participants signed an informed consent before being interviewed. The interviewer (KJC) knew two of the ten participants prior to interviewing them, having been their co-worker previously (with no power relationship) and they were fully aware of his identity prior to consenting. It appeared that this shared experience put the participants at ease during the interviews. To ensure participants could be open and transparent about their work context without risk of there being any repercussions, we removed any locality-specific data from all study reports.

### 2.4 Data analysis

Data analysis used an inductive approach (Thorne, 2016) to thematic analysis (Braun & Clarke, 2006). The initial focus was on familiarization with the data and gaining an understanding of the way each participant interpreted their role, work environment and stress in general. Once an understanding of a participant’s background was formed, the primary author (KJC) undertook an iterative process of data coding,

Throughout the process, codes were reviewed multiple times by all authors. If necessary, codes were refined with the aim of letting them remain open, short and close to the data. To avoid too much focus on the “micro” detail in the data, the coding process was also guided by Hunt’s (Hunt, 2009) recommendation, where line-by-line coding was conducted with some general questions in mind. This included: What is happening here? What does this mean? What are we learning here? What is this saying about work stress? The initial line-by-line coding was followed by coding larger extracts of data, which allowed further refinement of codes and helped in naming a number of code categories. Following coding each participant’s transcript, analysis moved to comparing codes and categories across the data from all participants. This was an iterative process and was continued throughout the data analysis as each new data set was collected.

The coding was followed by a process of identifying, analyzing and interpreting themes within data. Themes can represent a pattern or meaning within the data set (Braun & Clarke, 2006). The codes and categories that were identified during coding were organized into potential themes along with relevant data extracts. Candidate themes were discussed with NMK and KMM to ensure they were consistent with participants’ stories captured within the dataset. All proposed patterns and relationships were challenged with two main questions in mind: “What am I seeing here?” and “Why am I seeing that?” (Thorne, 2016). NMK and KMM feedback informed refinements to the development of themes. Any disagreements were resolved in discussion. During this process, themes were clearly defined and named. Themes identified in thematic analysis guided the write-up of study results. The final themes are reported below, along with illustrative interview extracts.

### 2.5 Rigour

To ensure scientific rigour, we followed Thorne’s (Thorne, 2016) principles for qualitative research: epistemological integrity, representative credibility, analytic logic and interpretive authority. To ensure *epistemological integrity*, we followed the methodological guidelines for Interpretive Description (Thorne, 2016). Regarding *representative credibility*, we applied purposive sampling to seek diversity in key characteristics, and used a combination of different analysis methods to strengthen the analysis process. Inclusion of raw data in the findings section enables the reader to evaluate the credibility of interpretation themselves. Regarding *analytic logic and interpretive authorit*y, prior to starting participant recruitment, the primary author (KJC) made a written reflection on their pre-existing assumptions about support-workers and their work stress due to the fact that the researcher had worked as a support-workers themselves. Their key assumptions included: caregiving is stressful; stress has a negative impact on people and needs to be minimized; stress was associated with a feeling of inadequacy, i.e. inability to deliver the quality care one expected. Finally, to reflect the *interpretive authority* principle, the data was interpreted considering this study’s context (see Table I), and no a priori codes were used.

**Table I.** Participants’ demographic characteristics.

Pseudonym	Gender	Age range ^a^	Country/region of origin ^b^	Years of experience	Educational attainment
Helen	F	50–59	Pacific Islands	4	Tertiary
Valerie	F	30–39	Asia	2	Tertiary (health-related)
Patricia	F	30–39	North America	7	Tertiary
Jim	M	50–59	NZ	6	Secondary
Amanda	F	50–59	Pacific Islands	16	Secondary
Rosie	F	60–69	NZ	16	Tertiary
Stacey	F	30–39	NZ	2	Secondary
Olivia	F	20–29	Asia	1	Tertiary (health-related)
Diane	F	40–49	NZ	4	Secondary
Lana	F	30–39	South America	2	Tertiary

^a^Age range rather than specific age is reported in order to protect participants’ identity.

^b^For people not born in NZ, region of origin is reported rather than specific ethnicity, in order to protect their anonymity.

## 3. Findings

Fifteen of the twenty-one people who expressed interest met the study eligibility criteria. One person who originally agreed to take part, refused on the day of their interview, without giving a reason. Eventually, ten participants were included and interviewed in this study.

Table I presents the demographic characteristics of the consenting participants using pseudonyms to protect anonymity. The study sample includes participants from a diversity of age groups, ethnicities and years of experience. As such, the sample allowed exploration of a breadth of experience.

In general, stress appeared to be both a mental and a physical experience. In some cases, it seemed to help people “get things done faster”, but mostly participants reported it had a negative impact on them and their ability to provide quality care.

“Your mind is in too many things at the time. You are sweating, rushing … because of the rushing you are not able to give quality of work in those tasks. You are multitasking, and you are not able to give quality to each one.” (Olivia)

**Figure 1.**
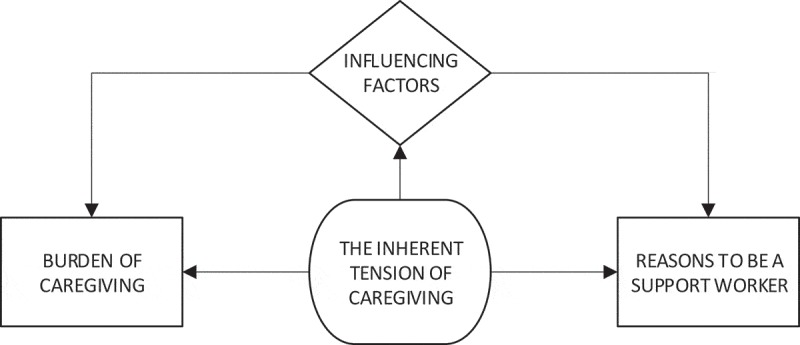
The inherent tension of caregiving.

Work stress was conceptualized by participants as being an everyday experience of having too much to deal with and feeling under constant pressure. It appeared to be a complex and fluid experience representing an inherent, dynamic tension between reasons to be a caregiver and the burden of caregiving (Figure 1). Participants also highlighted a range of influencing factors which may account for that fluidity. These findings are discussed below with supporting quotes.

### 3.1 The inherent tension between the reasons to be a caregiver and burdens of caregiving

The experiences described by the participants appeared to revolve around the reasons to be a caregiver and the burden of caregiving. The coexistence of these two aspects represented the tension inherent in caregiving, giving rise to participants work stress experiences.

#### 3.1.1 The reasons to be a caregiver

The participants of this study seemed to have an immense sense of empathy and wanted to see the care recipients happy. Driven by their commitment to care, they often did “their utmost best” to help them live the best life possible. For many, this was the main reason to keep doing what they do.

“I just want to say, no matter what I say about my stress, I love what I do, I am really passionate about the people I care for and I work with my heart.” (Helen)

Participants felt accountable for what they were expected to do by management, however, their sense of responsibility appeared to go beyond that. They felt responsible not only for the people they directly worked with, but for all care recipients within their facility.

“Here I am on a day off, going completely and utterly out of my way to make sure that this person I don’t even work with, I don’t even support him in the facility, but here I am with that stress of making sure he gets home.” (Diane)

Some participants simply reported needing a job to be able to pay their bills and provide for their families. For both Lana and Valerie, who were new immigrants, caregiving was their first job in NZ. They “really needed a job” and “had no other choice” but to become caregivers.

#### 3.1.2 The burden of caregiving

While participants took their jobs very seriously and appeared to do whatever it took to “give their clients a quality of life”, there was a downside to this deep personal commitment and sense of responsibility. Patricia suggested that what she was actually doing as part of her caregiving role was more than her job description suggests.

“It’s a huge amount of work for people to turn around and say: that’s actually your job … which I know is my role, but most of the time we go over to what’s on the piece of paper.” (Patricia)

Furthermore, it was apparent that the majority of participants struggled to keep boundaries around their work and it often affected their personal lives. Lana stated that she “had no life”.

“I was working during the week, weekends. Swap sometimes for night time. Sometimes I was working 3pm to 11pm, and the next day you had to start 7am. Do till 3pm. And come back again. All like that. Weekends … I had time for nothing, to have an ice cream, go out with my daughter, or with my family … ” (Lana)

While participant’s appeared to expect themselves to go above and beyond and be able to give the care recipients the best lives possible, in reality, there appeared to be a number of costs and consequences they had to consider. It was this conflict between the reasons to be a caregiver and the burden of caregiving that seemed to be at the heart of their work stress experience.

### 3.2 Factors influencing work stress experience

There were a range of factors which were perceived to influence the experience of work stress relating to person context, work environment and coping strategies.

#### 3.2.1 Person context

Participants reported there were a number of personal skills which were important. For example, Jim suggested that one of the crucial skills a caregiver needed was to be able to listen and “pick out the real problems instead [of] all the ones that are running through [patients’] heads”. Being able to understand what was really bothering the people they were caring for helped identify the triggers for their behaviors and avoid unnecessary stressful situations.

English as a second language also seemed to play an important role. It appeared stressful for both caregivers for whom English was a second language and for other caregivers who had to communicate with them. When Lana first started working as a caregiver, her command of English was very poor. She struggled to understand what was being said at handovers and what she read in medical notes:

“I look at the person, what he has, and then this is for me like Japanese, because I understand nothing”. (Lana)

The language barrier made her “want to cry” and was “hard and stressful”. Not knowing the language and being new in the country appeared to make her feel lost and vulnerable. “It’s like you are born again”, she added.

Cultural background appeared to play an important role in how caregivers perceived their relationship with care recipients. Olivia, who was born and raised in India, observed the relationship between a health care professional (and carer) and a care recipient in NZ was “very different”. She was used to a more authoritative relationship and she felt that the people she cared for did not have respect for her.

“Here the doctor has to ask: can I give this or can I do that? It’s very different in India. The doctor would just say: have this or I am going to give you an injection. You know, the authority thing (…). There is no respect, there is no sense of authority.” (Olivia)

#### 3.2.2 Work environment

Participants discussed the role of team dynamics in their work. Olivia felt that she worked in a “big team” and that they had “a good social thing” where staff could talk to each other if they got stressed. Socializing with her co-workers was also valued by Stacey. It allowed her to “destressify” with people who know her and she cared for, and understood the reality of her work.

Rewarding and acknowledging caregivers for their efforts seemed to have a positive impact on their work and attitude. Being rewarded for her hard work appeared very important to Lana. It gave her motivation to work and enhanced her confidence.

“Then I got one certificate—‘caregiver of the month’. And so: I did it!” (Lana)

Given caregivers are the people who spend the most amount of time with residents compared to other team members, participants argued that it seemed reasonable to include their views and opinions about a care recipient in the care planning process. For the participants of this study, the reality was different and it made them feel undervalued.

“Everyone here will go, and the key worker. We are never invited to any of that stuff, we don’t have a say. (…) They see them for maximum 5 hours in a week. We see them a minimum 40  hours in a week. Minimum 40 hours. And it can be really frustrating that our opinions don’t count.” (Stacey)

Participants also commented that their low wages contributed to their sense of feeling unimportant and undervalued. Adding to this, they did not see themselves as being in a position to negotiate, because they could be easily replaced by someone else “who really needs the job”.

“And the management of the facility doesn’t care if you’re tired, if you have stress. Because if you don’t want to work, they say—‘If you don’t want to stay here, then leave. We will take another one, who really needs the job.’” (Lana)

As a consequence of staffing difficulties, many participants reported being asked to perform additional roles for which they were not trained, or asked to “do a job that is not their job”. Participants felt stressed about this, particularly when asked to do things they did not feel competent in, for example, giving out medication.

Participants also reported feeling that when things went wrong, a care recipient or their family would always be perceived by management as having a more legitimate view than the support-workers’. Diane recalled a situation which involved one of her work colleagues, and found it to be very stressful for the worker and herself.

“I’ve actually witnessed jobs’ been lost because of service users and what they’ve said. And the fact that they have been believed over the staff. Last year as a union delegate I had to support a woman who was accused of passing herself off as a family member of the service user, which she didn’t. This service user was saying: ‘oh, but she did!’. And there was another staff member on who backed the service user up with that.” (Diane)

#### 3.2.3 Coping strategies

A number of participants reported that when dealing with a stressful situation at work they would simply shut off and not engage with the care recipients. Patricia believed that a caregiver needed to be able to draw a line between themselves and the person they cared for:

“If you keep engaging with the person it’s gonna hurt you more. It’s like putting a line, drawing a line, that’s ok. And then you become kind of cool.” (Patricia)

For Valerie, not engaging was part of being a professional. She consciously avoided engaging with the care recipients as people, not going beyond the requirements of the task at hand because it helped her deal with work stress.

“We just do our work and we are checking ourselves that our work is done properly. That we are doing the right things for him. Without any emotions. Just dealing as professionals.”(Valerie)

According to Diane, one had to acknowledge the difficulty and deal with a situation in a conscious way. She explained that this was particularly important in caregiving where one did not have “the luxury” to ignore a care recipient who needs help, but causes stress.

“Once I’ve acknowledged it, I work out (…) basically if I can do something about it, I will. If I can’t, that’s where the laid back bit comes in I think. Just: well, I can’t do anything about that, and just go on and see what’s gonna happen.” (Diane)

Importantly, all participants who had tried counselling were very satisfied, saying it was a great experience and a really effective way of dealing with their work stress. It was a tipping point for Lana, who at the time was also dealing with a difficult personal life situation.

“All the guys there, some of them didn’t want to talk about their lives. But I didn’t want to, but I was in such a situation and I had to sit down cry and say my story. I said: ‘please help, what do I do? I have to look after my daughter, what do I want for her? How do I go out of the grief?’ Every week she was talking about different topics and it helped me.” (Lana)

## 4. Discussion

This study highlighted that for many caregivers, the experience of stress was both fluid and complex. Findings suggest that support-workers may be torn between the reasons to be a caregiver and the burden of caregiving, with this in turn contributing to experiences of work stress. Importantly, the impact of stress on the workers and their work appeared to depend on a range of potentially modifiable factors, which can be grouped as person context factors, work environment factors, and coping strategies. This section starts by discussing the inherent tension in caregiving in the context of current research. It then focuses on a selection of key findings that have the potential to advance research. Finally, the study’s limitations are discussed.

### 4.1 The inherent tension in caregiving

The reasons to be a caregiver highlighted in this research related to either needing a job or a commitment to care. A number of study participants reported that they needed a job to help them “make ends meet”. This notion is perhaps unsurprising and evident in other research. As suggested by Sung, Chang, & Tsai, 2005 in their study involving nurses’ aides, monetary needs are an important reason for continuing to work as a support-worker. Clarke (Clarke, 2001), in her Canada-based study, noted that the support-workers workforce included highly qualified immigrants from the Philippines and Eastern-block countries, and that many of them did this job simply to meet their economical obligations. Similarly, a number of the current study’s participants were new NZ immigrants, and even though they had bachelors or higher degrees, they felt they “had no other choice” but to become caregivers in order to generate an income.

However, for many participants in the current study, being a support-worker was not only a source of income, but also meant doing something worthwhile. They described their belief that they were helping vulnerable people and were making a positive change in their patients’ lives. Most participants talked about their job and patients with passion, a sense of commitment and considered their work very rewarding. This is consistent with the current literature. Häggström, Skovdahl, Fläckman, Kihlgren, & Kihlgren, 2004 and Ravenswood et al., 2014 found that many support-workers reported “love” for their job and felt that they got a lot back from their work. Other studies found that the caregivers felt “needed” by the patients (McCluskey, 2000; Sung et al., 2005) and that they did “something worthwhile” (Atchison, 1998).

The burden of caregiving appeared to arrive through a clash between the commitment to care and the caregivers’ work reality. The findings suggest that for many participants the patients’ well-being was the highest priority. Many participants reported constantly feeling responsible for their patients, and in some cases even for all patients at their facility regardless of whether they were at work or not. As discussed by Häggström et al., 2004, a perceived sense of absolute responsibility can be a source of “irritation”, and become a major burden for the support-workers. With workers aiming to maximize their patients’ quality of life, such a sense of responsibility may lead to feelings of dissatisfaction and of failing their patients (Häggström et al., 2004).

The perceived and assumed responsibility in caregiving has been highlighted as an important issue in the recent literature (Ahlström & Wadensten, 2012; Elwér, Aléx, & Hammarström, 2010; Gustafsson et al., 2008; Häggström et al., 2004). Elwér et al., 2010 argued that caregiving is historically associated with responsibility. However, given that the pressure on resources is increasing (Blendon & DesRoches, 2003) while the demand for and on healthcare is growing, the strong sense of responsibility for meeting the demands of caregiving has become problematic, and is likely to become even more so. Support-workers’ attempts to meet these increasing demands may lead to increased levels of work stress and consequently decreased quality of care.(Hayes et al., 2012).

The burden on support-workers appeared to have negative consequences. Participants in the current study commonly reported feeling mentally and physically tired. Fatigue and burnout have been reported to be a common problem for support-workers (Hooper, Craig, Janvrin, Wetsel, & Reimels, 2010; Kennedy, 2005; Santana et al., 2013). This may negatively affect caregivers’ well-being and has been found to be associated with other issues such as patient abuse (Cohen & Shinan-Altman, 2011; Shinan-Altman & Cohen, 2009).

### 4.2 Factors influencing support workers’ work stress experience—key findings and implications

The analysis highlighted a range of potentially modifiable factors influencing the stress experience. This section will focus on a selection of key findings: lack of recognition, unintended consequences of “person-centred care” and specific caregiving challenges resulting from reliance of healthcare systems on migrant workers.

#### 4.2.1 Lack of recognition

One of the key findings of this study was the perceived lack of recognition of what support-workers do in their role and of what they could be contributing as part of the health care team. The first notion (“role recognition”) refers to the fact that caregivers have an extreme sense of emotional connection to their caring role and frequently go above and beyond their formal job expectations. The latter (expertize recognition) relates to caregivers’ feelings of not being heard, and that despite having important expertize to offer the healthcare team, their knowledge is not respected. Lack of recognition has been identified as an important work stressor for support-workers previously (Clarke, 2001; Gustafsson et al., 2008; Häggström et al., 2004; Hertting et al., 2005; Ravenswood et al., 2014; Secrest et al., 2005). It refers to recognition of the workers’ skills, capabilities and knowledge; of the quality of care they provide and the relationships they develop with the care recipients; and of the overall role they play in the health care team and system. Support-workers want to be recognized and valued for what they do (Sung et al., 2005), and it is crucial for the organizations and broader structures in which they work to help address this issue.

Participants of this study suggested that one way of increasing the recognition of their role that would also potentially enhance team building, would be for management staff to occasionally (e.g. once per year) buddy-up with the caregivers for some time and accompany them throughout the shift. Such a strategy could expose the managerial staff to the challenges support-workers face daily. In turn, it may validate the workers’ feelings of struggle and potentially help them deal with the difficulties they experience. Other strategies that could increase the recognition of their role in the healthcare system include: written acknowledgement (Cronin & Becherer, 1999), awards (Frey & Gallus, 2014) and provision of training and upskilling opportunities (Voegtlin, Boehm, & Bruch, 2015).

Another way of providing recognition would be by increasing remuneration and this is a widely debated topic around the world (Health Education England, 2015; Parsons, Simmons, Penn, & Furlough, 2003; Ravenswood et al., 2014). Cronin and Becherer (Cronin & Becherer, 1999) found that monetary recognition received the top ranking in their survey of nursing staff’s meaningful types of recognition. However, remuneration is clearly not the only option (Cronin & Becherer, 1999; Naeem & Zaman, 2013). In the current study, it was noteworthy that recognition would come (or be noted as absent) in many forms—particularly in how the knowledge or perspective of the support-worker was considered (or ignored) by qualified health professionals.

Regardless of the outcome of funding debates, careful consideration of the role of support-workers in the healthcare system is required. The potential impact of the care they provide on the quality of life of many people requiring long-term care is significant and growing. Our findings suggest support workers have much to offer with regard to improving long-term care, given their first-hand knowledge of their patients and their direct daily contact with them. They could be very effective mediators in the process of long-term care. However, first they will have to be recognized as legitimate members of the health and social care team.

#### 4.2.2 Unintended consequences of “person-centred care”

The participants of this study appeared to have a huge sense of empathy and were driven by a commitment to care. They strived to help the care recipients live the best life possible. However, an interpretation of patient-centered care as simply meaning the “patient is always right” seemed to disempower the support-workers. As noted by Brooker (Brooker, 2003), the confusion around what patient-centered care means might be one of the reasons for the abovementioned disempowerment. A simplistic understanding is that it is about seeing patients (or care recipients) as experts and putting them at the center of decisions (McCance, McCormack, & Dewing, 2011). Participants of the current study reported feeling that a care recipient or their family would be always believed over a support-worker. This demoralizing experience for these workers were reported to contribute to feelings of being unheard and at the bottom of the work-force hierarchy. However, truly patient-centered care values both the care recipients and the people who care for them (Brooker, 2003). Thus, perhaps more aptly called person-centered care, support-workers who provide the majority of care to residents in long-term care facilities (Health Education England, 2015; McCance et al., 2011) should be recognized and considered as persons/people, and valued more than they currently appear to be. Moreover, they need to be supported to provide care that is person-centered even when, or perhaps particularly when, this is difficult.

#### 4.2.3 Specific challenges resulting from healthcare system reliance on migrant workers

A large proportion of the current study’s sample were not born in NZ (see Table I), reflecting the global context (including in Australia, Ireland, UK and USA), where there is a growing reliance of healthcare systems on migrant workers (Ravenswood et al., 2014; Walsh & O’Shea, 2010). Many of these workers use English as their second language and are not familiar with local culture of the countries they work in (Walsh & O’Shea, 2010). The significance of delivering culturally appropriate care is particularly relevant in NZ where the indigenous population (Māori) have a strong influence on culture and what is considered appropriate care (Came, Cornes, & McCreanor, 2018), and where reliance on migrant workers is high (Ravenswood et al., 2014). Despite the long-standing recognition of the importance of culturally appropriate care (Williamson & Harrison, 2010), more research is required in this area (Degrie, Gastmans, Mahieu, de Casterlé, & Denier, 2017; Stone, 2016; Williamson & Harrison, 2010). The findings of the current study indicate that cultural appropriateness should also be considered in the context of the support-workers cultural needs. Thus, further research investigating strategies facilitating adjustment of workers of different cultural backgrounds is recommended.

For many migrant workers (including participants in the current study), poor command of English language leads to difficulties with communication, and impacts the quality of care for care recipients (Walsh & O’Shea, 2010). The findings of the current study highlighted that speaking English as a second language may play an important role in work stress and act as a stressor itself. Being unable to communicate efficiently was problematic for those where English was their first language, as well as those who struggle with English. The impact of communicating in a second language on stress has been long recognized in psychology (Peck, 1974; Statistics New Zealand, 2013). Recent NZ Census data, suggests that English may be a second language for at least 10% of NZ’s total population. This is in line with other countries who rely heavily on migrant workers to deliver healthcare (Australian Bureau of Statistics, 2017; Ryan, 2013). At the same time, no caregiving-related studies investigating this important issue in NZ have been identified. Such studies would be particularly beneficial in the bilingual and multicultural country like NZ, where many support-workers as well as care recipients do not communicate primarily in English.

### 4.3 Limitations

This study provides an in-depth exploration of NZ support-workers perspectives and provides valuable insights into the experience of their work stress. The use of purposive sampling facilitated inclusion of participants from a range of demographic groups. As work stress has not been explored in this population before, this study is a valuable addition to the body of knowledge about work stress in NZ support-workers.

The findings of the study need to be considered in the context of its limitations. The recruitment was conducted in an urban, ethnically diverse city. It is possible that the experiences of support-workers in smaller communities or different settings may offer some alternate perspectives to this topic. Future research could build on the current study to explore experiences in a wider range of settings, contexts and geographical locations.

It should be noted that study findings are specific to the participants’ experiences and may not be broadly generalized beyond this population. This study employed an Interpretive Descriptive approach (Thorne, 2016), and there may be some commonalities and shared experiences for other support-workers in similar situations.

## 5. Conclusion

Support-workers experience of work stress represented an uneasy tension that varied and was complex by nature. This study enhances understanding of support-workers experience of work stress by highlighting the challenges relating to the lack of recognition of support-workers’ role and expertize, the unintended consequences of person-centered care, and the challenges faced by migrant support-workers in many healthcare systems worldwide. These findings, within the context of increasing pressures within the health care system, indicate work stress in support-workers warrants further attention in research and practice. The findings could be used to guide the development of interventions aiming to improve both the work environment (role recognition, peer support, teamwork) and the caregivers’ ability to cope with stress.
